# Consistency of Financial Interest Disclosures in the Biomedical Literature: The Case of Coronary Stents

**DOI:** 10.1371/journal.pone.0002128

**Published:** 2008-05-07

**Authors:** Kevin P. Weinfurt, Damon M. Seils, Janice P. Tzeng, Li Lin, Kevin A. Schulman, Robert M. Califf

**Affiliations:** 1 Duke Clinical Research Institute, Duke Translational Medicine Institute, Duke University School of Medicine, Durham, North Carolina, United States of America; 2 Department of Psychiatry and Behavioral Sciences, Duke University School of Medicine, Durham, North Carolina, United States of America; 3 Department of Medicine, Duke University School of Medicine, Durham, North Carolina, United States of America; University of Exeter, United Kingdom

## Abstract

**Background:**

Disclosure of authors' financial interests has been proposed as a strategy for protecting the integrity of the biomedical literature. We examined whether authors' financial interests were disclosed consistently in articles on coronary stents published in 2006.

**Methodology/Principal Findings:**

We searched PubMed for English-language articles published in 2006 that provided evidence or guidance regarding the use of coronary artery stents. We recorded article characteristics, including information about authors' financial disclosures. The main outcome measures were the prevalence, nature, and consistency of financial disclosures. There were 746 articles, 2985 authors, and 135 journals in the database. Eighty-three percent of the articles did not contain disclosure statements for any author (including declarations of no interests). Only 6% of authors had an article with a disclosure statement. In comparisons between articles by the same author, the types of disagreement were as follows: no disclosure statements vs declarations of no interests (64%); specific disclosures vs no disclosure statements (34%); and specific disclosures vs declarations of no interests (2%). Among the 75 authors who disclosed at least 1 relationship with an organization, there were 2 cases (3%) in which the organization was disclosed in every article the author wrote.

**Conclusions/Significance:**

In the rare instances when financial interests were disclosed, they were not disclosed consistently, suggesting that there are problems with transparency in an area of the literature that has important implications for patient care. Our findings suggest that the inconsistencies we observed are due to both the policies of journals and the behavior of some authors.

## Introduction

The influence of commercial interests on medical science is an area of ongoing concern. Recent work has examined the extent of this influence [1]–[5] and explored best practices for managing conflicts of interest [6]–[9]. A particular area of concern is the influence of conflicts of interest on the biomedical literature. Disclosure of authors' financial interests in journal articles has been proposed as one strategy for protecting the integrity of research and maintaining public trust [10]–[12]. The International Committee of Medical Journal Editors (ICMJE) and the World Association of Medical Editors, among others, encourage disclosure of authors' financial interests [10], [13]–[15].

Despite repeated calls for financial disclosures and the growing number of journals with conflict-of-interest policies [12], [16]–[18], the effectiveness of current approaches to disclosure remains unclear [16], [19]–[23]. How one defines effectiveness may depend on the parties involved, be they authors, editors, reviewers, or readers. For readers of the medical literature, it might be reasonable to expect that authors' financial interests are disclosed in similar ways in articles on the same topic published around the same time. A system of disclosure that was not consistent in this way might create confusion or mistrust among readers and the public. In this study, we examined all articles on coronary stents published in 2006 to determine whether authors' financial interests were reported consistently.

A coronary stent is a flexible metal tube inserted into a coronary artery to hold the artery open. Stents are widely used in patients with coronary disease to restore or maintain blood flow to the heart muscle, which is critically dependent on high rates of blood flow. Coronary stents have come under intense scrutiny, including disagreements about their effectiveness [24], controversy in the United States regarding an early decision to adjust Medicare payments for drug-eluting stents [25], and concerns about the financial interests of US Food and Drug Administration advisory panel members [26]. Messages that appear in the medical literature regarding the effectiveness of stents affect a multibillion-dollar industry that includes companies that produce stents and products that support stents, as well as companies that produce alternatives to stents. The integrity of this literature is important for disseminating accurate information about the risks and benefits of stents. The controversy, financial stakes, and impact on patients' welfare make this an appropriate literature to study regarding the disclosure of potential conflicts of interest.

## Methods

### Data Sources

In March 2007, we searched PubMed for English-language articles published in 2006 and indexed with the Medical Subject Heading (MeSH) terms “stents,” “heart diseases,” and “humans.” We limited the search to articles published in 2006 because this was the most recent year for which data were available; and we limited the search to a single year on the assumption that financial interests change over time and would be more or less stable within a year. Two of us independently reviewed the articles and selected those that provided evidence or guidance regarding the use of coronary artery stents. The reviewers reconciled their determinations by consensus, and a third reviewer resolved disagreements and uncertainties. We excluded 2 articles for which no authors were listed.

Because many author names were associated with multiple articles, we sought to ensure that each author in the database was represented by a single term. First, we combined authors who were indexed in Medline under different names for different articles. For example, if we determined that author “Smith J” in one article and author “Smith JA” in another article were the same person, we marked both instances as “Smith JA” in the database. Next, we separated different authors who were indexed in Medline under the same name. For example, if we determined that author “Jones A” in one article was not the same person as author “Jones A” in another article, we marked one author as “Jones AA” and the other as “Jones AB.”

We constructed a Microsoft Access database for the analysis that contained the complete Medline citation for each article, including author names, publication types, and MeSH terms. We recorded whether each article contained a statement regarding financial or material support for the work; sources and types of support; and whether the article described authors' contributions to the work. If the article described authors' contributions, we recorded each author's contributions. If the article contained a statement regarding authors' financial interests, we recorded the types of interests and the organizations with which each author had the specified relationships. If the article directed readers to supplemental disclosure information located elsewhere (eg, the journal's Web site), we included the supplemental information in our coding.

We consulted ISI Journal Citation Reports to determine journal impact factors as an indicator of the influence of each journal [27]. We used the 2005 impact factor for each journal (the most recent available at the time of the study), defined as the average number of times that articles published in the journal in the previous 2 years were cited in 2005. We also determined whether representatives of each journal requested listing as endorsers of the ICMJE guidelines, as reported by the ICMJE [28].

### Statistical Analysis

We categorized articles as “research” if they reported original research or a case study. We categorized the remaining articles as “other,” which included mostly commentaries and letters. We report simple frequencies and percentages for whether sources of funding were disclosed for research articles, excluding case reports. (Because case reports are drawn from clinical practice, we reasoned that no funding source statement would be expected.) We used χ^2^ tests to examine relationships between reporting a source of funding and endorsement of the ICMJE guidelines. We used a Wilcoxon rank sum test to examine the relationship between reporting a source of funding and the impact factor of the journal in which the article was published.

If an article contained a statement describing an author's financial interests (including a declaration of no interests), we considered a disclosure statement to be present. We coded each article as having disclosure statements for all, some, or none of the authors. We used χ^2^ tests to examine the relationships between the presence of disclosure statements for authors (all, some, or none) and type of article and endorsement of the ICMJE guidelines. We used a Wilcoxon rank sum test to examine the relationship between the presence of a disclosure statement for authors (all vs some or none) and the impact factor of the journal in which the article was published.

### Prevalence and Nature of Disclosures

We examined the prevalence and nature of authors' financial disclosures by analyzing every instance in which a disclosure might have appeared (hereafter termed *author instances*). The number of author instances is the sum of the number of authors from all articles in the database. For example, if someone authored 6 articles, that person contributed 6 author instances to the data set. Across author instances, we examined the presence or absence of a disclosure statement. We used χ^2^ tests to compare the frequencies of disclosure statements between research articles and other articles. We conducted the same analysis after removing case reports from the research category.

### Consistency of Disclosures

For authors with more than 1 article, we analyzed the consistency with which disclosure statements appeared across multiple articles. We conducted this analysis in two ways. First, we determined whether there was consistency within author in the presence or absence of any type of financial disclosure statement. Second, for authors who disclosed at least 1 relationship with a commercial organization, we evaluated the consistency within author of the content of the disclosures. We describe each of these analyses in turn.

#### Consistency in the Presence or Absence of Disclosure Statements

We designed this part of the analysis to identify authors whose articles always, sometimes, or never agreed in terms of the presence or absence of a disclosure statement. Thus, for each author, we estimated the proportion of all unique pairwise article comparisons that agreed in terms of the presence of a financial disclosure, a declaration of no interests, or an absence of any disclosure statement. (Note that for these analyses, 2 articles by the same author in which 2 different organizations were disclosed would be counted as agreement. A later analysis addressed consistency in the organizations disclosed.) For example, consider an author of 4 articles with the following disclosures: article 1 (relationship with a company), article 2 (relationship with a company), article 3 (declaration of no interests), article 4 (no disclosure statement). In this case, there are 6 unique pairwise comparisons, and 1 pair is in agreement (articles 1 and 2). Therefore, the proportion agreement for the author is 1/6 (0.17). We report the frequencies of authors with proportion agreement equal to 0 (no articles agree), 1 (all articles agree), and between 0 and 1 (some articles agree).

We also examined the frequency of types of agreement (present–present, none declared–none declared, absent–absent) and disagreement (present–none declared, present–absent, none declared–absent). Due to the small average number of articles per author, we collapsed these types of agreement across all comparisons for all authors. To examine how disagreements might be a function of journal policies, we report whether the disagreements occurred within the same journal and whether they occurred within the same article type (research–research vs research–other). Because some readers might consider listing an author's affiliation with a company as a disclosure (in lieu of a specific disclosure statement), we determined the number of industry-affiliated authors contributing to each type of agreement and disagreement.

#### Consistency in the Content of Disclosure Statements

To determine the consistency of authors' disclosure statements, we examined authors who had multiple articles and disclosed at least 1 financial interest. For each of these authors, we first counted the number of unique organizations with which the author disclosed a relationship in any of the author's articles. (In some cases, a relationship with more than 1 organization was disclosed in a single article.) Consider a hypothetical example in which an author has 4 articles with the following disclosures: article 1 (relationship with company A), article 2 (relationships with company A and company B), article 3 (no disclosure statement), and article 4 (relationship with company A). This author has 4 articles in which 2 unique organizations—companies A and B—appear in disclosure statements. Next, for each of the unique organizations associated with an author, we counted the number of organizations that were disclosed in all of the author's articles. In the hypothetical example, company A is disclosed in 3 of the 4 articles and company B is disclosed in 1 of the 4 articles. Thus, none of this author's organizations are disclosed in all 4 of the articles. For each author, we calculated the proportion of unique organizations with which the author disclosed a relationship that were disclosed in each of the author's articles, and we summarized the distribution of these proportions across authors.

## Results

We retrieved 746 articles, of which 623 were research articles (eg, clinical trials, case reports, meta-analyses) and 123 were commentaries, letters, and other communications that did not present original research. There were 2985 authors and 135 journals. The number of articles per author ranged from 1 to 25.

Of the 441 research articles (excluding case reports), 316 (71.7%) did not include a statement identifying the source of support for the study (including declarations of no support). These statements were more likely to appear in journals that endorsed the ICMJE guidelines (34% vs 21%; χ^2^ = 9.3; *p* = 0.002) and in journals with higher impact factors (median impact factor, 3.55 vs 3.06; *p* = 0.04). A total of 125 organizations were listed as sources of support. The top 5 sources (ie, Johnson & Johnson, Boston Scientific, CardioVascular Research Foundation [South Korea]; Ministry of Health and Welfare [South Korea]; and Guidant) accounted for 28% of the articles in which a source of support was declared.

Eight of the 623 research articles (1.3%) contained statements describing the contributions made by each author.

### Financial Disclosures

Table 1 shows the percentages of articles in which a disclosure statement was present for all, some, or none of the authors. Overall, 116 articles (15.5%) contained a disclosure statement for all authors, and 620 articles (83.1%) did not contain a disclosure statement for any author. Articles in journals that endorsed the ICMJE guidelines were more likely than other articles to have disclosure statements for all authors (25.3% vs 7.4%). Articles in which all authors had disclosure statements were more likely to appear in journals with higher impact factors (median impact factor, 11.63 vs 3.06; *p*<0.001).

**Table 1:** Articles with disclosure statements for all, some, or no authors.*

Characteristic	Articles (N = 746)			*p* †
	Disclosure statement for all authors	Disclosure statement for some authors	Disclosure statement for no authors	
Article type				0.05
All	116 (15.5)	10 (1.3)	620 (83.1)	
Research	88 (14.1)	9 (1.4)	526 (84.4)	
Other	28 (22.8)	1 (0.8)	94 (76.4)	
Journal endorsement of ICMJE guidelines				<0.001
No	30 (7.4)	5 (1.2)	371 (91.4)	
Yes	86 (25.3)	5 (1.5)	249 (73.3)	

Abbreviation: ICMJE, International Committee of Medical Journal Editors.

* Data are expressed as n (row %) unless otherwise indicated.

† *p* values are from χ^2^ tests.

### Prevalence and Nature of Disclosures

A total of 168 authors (5.6%) had a disclosure statement in at least 1 article. The combination of authors and articles resulted in 4664 author instances (ie, opportunities for disclosure). Of these author instances, 220 (4.7%) had a disclosure, 577 (12.4%) had a declaration of no interests, and 3867 (82.9%) had no disclosure statement (Table 2). Author instances in research articles were slightly less likely to have a disclosure statement than author instances in other articles. Excluding case reports did not change the percentages by more than 1 percentage point (data not shown).

**Table 2:** Author instances with and without disclosure statements by article type (N = 4664).*

Disclosure statement	Article type†		
	All articles	Research	Other
Absent	3867 (82.9)	3629 (83.2)	238 (78.3)
Present			
Disclosure of financial interest	220 (4.7)	198 (4.5)	22 (7.2)
Declaration of no interests	577 (12.4)	533 (12.2)	44 (14.5)

* Data are expressed as n (%). The number of author instances is the sum of the number of authors from all articles in the database.

† *p* = 0.04 from χ^2^ tests for comparisons between research articles and other articles.

The maximum number of financial interests disclosed for a single author in a single article was 4. The disclosed relationships referred to 78 organizations. The 5 most frequent organizations (ie, Johnson & Johnson, Boston Scientific, Medtronic, Sanofi-Aventis, and Bristol-Myers Squibb) accounted for 59% of the relationships. In 4 cases, instead of a specific organization, the disclosure statement described relationships with multiple unnamed organizations (eg, “all drug-eluting stent companies,” “medical device companies” ). Of the 468 relationships disclosed, the most frequent relationship types included receipt of research support (25%), speaker fees (17%), and consultancies (15%).

### Consistency of Disclosures

Of the 2985 authors in the database, 683 (22.9%) authored more than 1 article and 307 (10.3%) authored 3 or more articles. For 454 of the authors with multiple articles (66.5%), each author's articles always agreed in terms of whether the articles disclosed a financial relationship, declared no relationships, or had no disclosure statement. For 86 of the authors with multiple articles (12.6%), there was no agreement among articles.

Table 3 shows the results of comparing the presence or absence of disclosure statements across articles for the same author. The large percentage of comparisons that resulted in agreement (4256/5573 [76.4%]) was due almost entirely to cases in which the absence of a disclosure statement was consistent across articles for the same author (4093/4256 [96.2%]). The most frequent cases of disagreement (840/1317 [63.8%]) occurred when one article contained a declaration of no financial interests and another article by the same author contained no statement. There were 26 cases in which one article disclosed an author's financial interests and another article declared that the same author had nothing to disclose. As shown in the last column and last row of Table 3, these 26 cases involved 16 authors. Authors with industry affiliations represented a small proportion (<12%) of any type of agreement or disagreement.

**Table 3:** Unique pairwise comparisons between financial disclosure statements in articles by the same author (N = 5573).*

Result of comparison	All comparisons (N = 5573)	Comparisons by whether the articles appeared in the same or different journals		Comparisons by article type combination			Number of authors represented	
		Different journals (n = 4219)	Same journal (n = 1354)	Research–research (n = 4919)	Other–other (n = 52)	Research–other (n = 602)	All authors	Industry-affiliated authors
Agreement	4256 (76.4)	2949 (69.9)	1307 (96.5)	3787 (77.0)	44 (84.6)	425 (70.6)		
Absent–absent	4093 (73.4)	2823 (66.9)	1270 (93.8)	3635 (73.9)	43 (82.7)	415 (68.9)	571	4
Present–present	96 (1.7)	67 (1.6)	29 (2.1)	87 (1.8)	0	9 (1.5)	34	4
None declared–none declared	67 (1.2)	59 (1.4)	8 (0.6)	65 (1.3)	1 (1.9)	1 (0.2)	40	0
Disagreement	1317 (23.6)	1270 (30.1)	47 (3.5)	1132 (23.0)	8 (15.4)	177 (29.4)		
Absent–none declared	840 (15.1)	825 (19.6)	15 (1.1)	729 (14.8)	7 (13.5)	104 (17.3)	168	1
Present–absent	451 (8.1)	420 (10.0)	31 (2.3)	380 (7.7)	1 (1.9)	70 (11.6)	69	8
Present–none declared	26 (0.5)	25 (0.6)	1 (0.1)	23 (0.5)	0	3 (0.5)	16	1

* Data are n (%) unless otherwise indicated.

As also shown in Table 3, comparisons between two articles in different journals were more likely to result in inconsistencies of disclosure than were comparisons between two articles in the same journal (30.1% vs 3.5%; χ^2^ = 420.0; *p*<0.001). In other words, almost all of the inconsistencies (1270/1317 [96.4%]) were between articles by the same author in different journals. Comparisons between research articles and other articles were more likely to result in disagreement than were comparisons between two research articles (29.4% vs 23.0%; χ^2^ = 12.1; *p*<0.001).

Among the 75 authors who disclosed a relationship with a specific organization, there were 2 cases (2.7%) in which the organization was disclosed in every article the author wrote about coronary stents in the same year.

## Discussion

Disclosure of authors' financial interests is thought to help preserve trust in the peer-review process and the credibility of research [13]–[15]. A decade ago, Krimsky and Rothenberg [29] encouraged journal editors “to take seriously the implementation of disclosure policies in response to the escalation of financial interests of authors.” Given the increasing concern regarding conflicts of interest in medicine, consumers of medical scholarship might reasonably desire a reliable system of disclosure in which journals and authors make disclosures appropriately and consistently. However, in this study of 746 articles on coronary stents in 2006, we found that the large majority of articles did not include information about funding sources and authors' financial interests. In the rare instances when authors' financial interests were disclosed, they were not disclosed consistently.

### Role of Journals

The inconsistency we observed may be due in part to journal practices. We found that 72% of research articles (excluding case reports) did not identify a funding source and that 83% of all articles did not have a financial disclosure statement for any author. Inconsistencies in financial disclosures for the same authors were due almost entirely to cases in which there was a disclosure statement in one journal and no disclosure statement in another journal. Together, these findings suggest that there are important variations among journals in the handling of information about research support and financial interests.

Some journals may simply not solicit or report information about authors' financial interests. Indeed, some journals in this study never published disclosure statements. Even among journals that sometimes published disclosure statements, we found inconsistencies. This finding is consistent with a recent study in which editors of journals with conflict-of-interest policies reported that they do not publish disclosure statements in every article [16].

Journal policies might vary in several ways. One issue concerns what journals report when authors declare that they have no financial interests to disclose. If every author who did not have a conflict of interest submitted a statement to a journal declaring no interests, our data suggest that variations in journal policies on this point could account for up to 64% (840/1317) of the inconsistencies we observed (see Table 3). Other differences in journal policies might account for the 34% of inconsistencies in which there was a declaration of a financial interest in one article but no disclosure statement at all for the same author in another article. For example, journals differ in how they describe the nature of the financial interests that authors should report (eg, “all financial and personal relationships that might bias their work,” [13] “any relevant financial interests,” [30] financial interests in any “private or public company in the health care field” [31]) or the relevant time period (eg, past 2 years vs past 5 years). Journals could also differ in terms of the criteria editors use to evaluate the risks posed by reported financial interests. Also, journals could differ in terms of whether and how they make disclosure statements available to readers. Finally, journals could differ in how they solicit and report disclosures for different types of articles. For example, some journals have different disclosure policies for commentaries and review articles than for other articles. It is unclear how journals and their editors determine policies relevant to these issues.

### Role of Authors

Some of the inconsistency we observed is likely also due to the behavior of authors. Only 6% of the 2985 authors in this study had an article that contained a statement about their financial interests, which suggests that many authors did not disclose their interests. Indeed, in recent cases, authors did not disclose financial interests that were later discovered by others [21], [32], [33]–[36]. In an informal Internet search on authors in our database for whom no interests were disclosed and for whom there were explicit declarations of no interests, we found evidence that some authors had relationships that many would consider to be conflicts of interest. These relationships included founding a company that manufactures stents, membership on advisory boards of stent manufacturers, and consultancies for stent manufacturers and companies that make drugs related to stent use. Thus, part of the problem with disclosure is the failure of some authors to disclose.

We also found 26 cases (involving 16 authors) in which authors disclosed a financial interest in one article but declared they had no financial interests in another article. (This is likely an underestimate of such contradictory statements, because some such authors might have declared they had no interests to a journal that does not report such disclosures, resulting in a “present–absent” comparison.) Almost all of the 26 cases occurred between two research articles, making it unlikely that these apparent contradictions were due to authors or editors believing that a financial interest was relevant for one type of article but not for another. These cases occurred infrequently, but the inconsistency is curious. Our data do not allow us to determine whether the authors intended to mislead.

Just as it is unclear how editors decide whether to publish disclosures, little is known about how authors judge the relevance of their financial interests and decide whether to disclose them. Many journal policies rely on authors to determine the relevance of their financial interests. There are at least two perspectives from which an author could judge the relevance of a financial interest. First, the author might try to judge the strength of influence they themselves perceive a financial interest to have. Although authors might have a unique vantage point, this type of introspection is limited, because people are often unaware of all of the determinants of their actions* [37], [38]. Second, an author might try to judge whether readers or the public would view a specific financial interest as relevant. One problem with adopting this perspective is that some authors might have difficulty predicting what readers would want to know. Based on guidelines and recent media attention [21], [33], [34], [39], a reasonable starting point for these authors might be to disclose relationships with stent manufacturers, companies making products that support stent use, and companies making alternatives to stents. An even more comprehensive strategy would require authors to disclose financial interests in any health care organization [31].

### Effects of Disclosure on Readers

Guidelines assume that a reader's discovery of previously undisclosed financial interests has detrimental effects on trust [13]–[15]. This is one consideration that has motivated calls for disclosure of authors' financial interests. However, little is known about how users of the literature and readers of media reports interpret disclosures and perceive authors' financial interests. If one purpose of financial disclosures is to allow readers to assess the influence of the financial interests on the overall study, presumably readers would also need to know the role played by the authors with the financial interests. However, we found that only 1.3% of research articles described the contributions of the authors.

It is unclear how readers' attitudes and behaviors would be affected if there were consistent and appropriate disclosures in the medical literature. Disclosures of commercial interests in research have elicited strong negative responses [25], [40], [41], whereas other work suggests that people do not take disclosures seriously enough [42], [43]. Studies of financial interests in the context of clinical research show that responses to financial interests are not straightforward and can depend on the type of interest involved [9], [44], [45]. More research is needed to provide a better evidence base for disclosure policies and to clarify how decision makers and the public use and perceive disclosures.

### Limitations

We necessarily restricted the study to articles in a single therapeutic area, so the results may not be generalizable to other areas. We do not have data that allowed us to trace for each article what information was solicited by the journal, what was reported by the author, and how disclosed information was processed by the editors. Moreover, we do not have knowledge of the financial interests that might have been relevant for authors at the time they submitted their manuscripts. Instead, the data we collected were from the perspective of the reader of the medical literature. More data are needed to elucidate the roles played by both journals and authors in producing the inconsistencies we observed.

### Conclusions

Our study is the first to examine the consistency of authors' financial disclosures in the biomedical literature. We have documented evidence of a systemwide problem with transparency in an area of the literature that has important implications for patient care. It could be argued that an inconsistent system of disclosure is more harmful than no disclosure at all. The current approach creates the impression rather than the reality of transparency and may encourage underestimation of the impact of conflicts of interest on the integrity of medical science.
